# Supplementary material for: Alternative transcription start site selection in Mr-OPY2 controls lifestyle transitions in the fungus Metarhizium robertsii
Source: Nat Commun. 2017 Nov 16;8:1565. doi: 10.1038/s41467-017-01756-1 (PMC5691130; doi:10.1038/s41467-017-01756-1)
Supplement: Supplementary file 2 — Supplementary Information [file 41467_2017_1756_MOESM2_ESM.pdf]

a

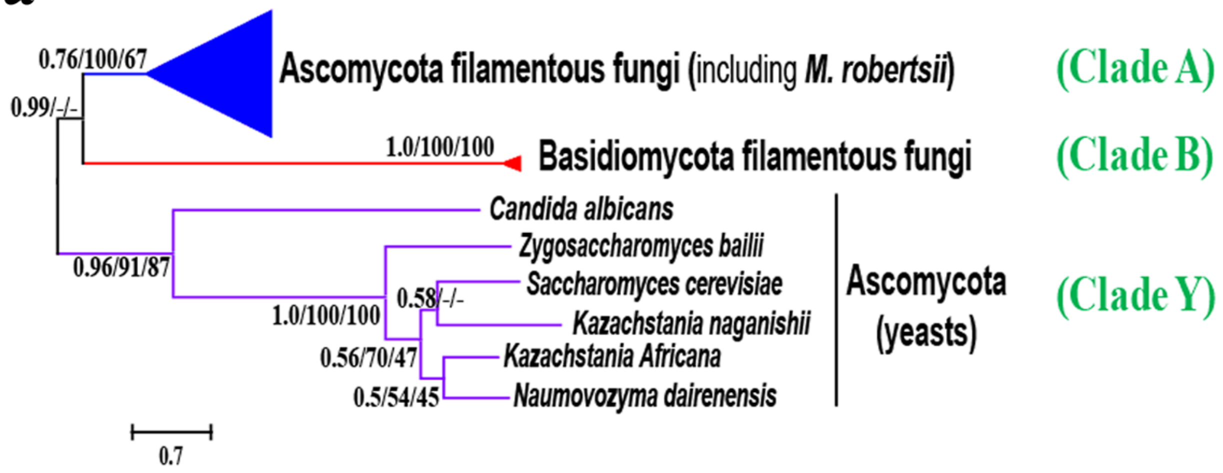

b

Statistics of comparison of topologies of alternative trees with the obtained tree (A)

Scenarios	obs	au	np	bp	pp	kh	sh	wkh	wsh
((A,B),Y)	-1.1	0.546	0.384	0.381	0.648	0.549	0.716	0.549	0.717
((A,Y),B)	1.1	0.483	0.320	0.322	0.224	0.451	0.622	0.451	0.621
(A,(B,Y))	1.6	0.425	0.301	0.296	0.128	0.428	0.589	0.428	0.589

Note: A: Ascomycota Clade; B: Basidiomycota Clade; Y: Yeast Clade

Supplementary Figure 1: Phylogenetic analysis of fungal OPY2 proteins. (**a**) Phylogenetic trees constructed with fungal OPY2 proteins. Numbers at nodes represent Bayesian posterior probabilities (left), bootstrap values of Neighbor-Joining (middle) and Maximum Likelihood (right). Hyphen (-) indicates no support value in the corresponding method. The scale bar corresponds to the estimated number of amino acid substitutions per site. Information about the sequences used in this analysis is presented in Supplementary Table 1. (**b**) Statistics of comparison of topologies of alternative (constrained) trees with the obtained tree (non-constrained tree presented in a). The clades contain Ascomycota filamentous fungi (A), Basidiomycota filamentous fungi (B), and Ascomycota yeasts (Y). The value of site-wise likelihood of every constrained topology was calculated by PhyML. Hypothesis testing was implemented with CONSEL. All tests consistently showed the obtained tree ((A, B), Y) is best supported than the constrained trees ((A, Y), B) and (A, (B, Y)).

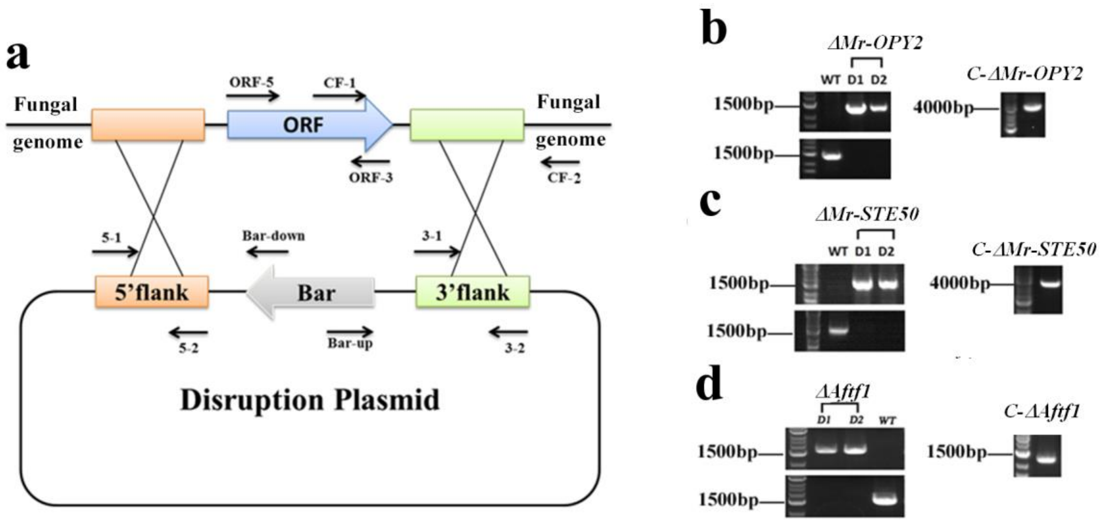

Supplementary Figure 2: Deletion of the *Mr-OPY2*, *Mr-STE50* and *Aftf1* genes in *M. robertsii*. **(a)** A schematic diagram of gene disruption based on homologous recombination showing a map of a disruption plasmid and its relative position in the fungal genome. **(b)** Confirmation of construction of the *Mr-OPY2* deletion mutant (*ΔMr-OPY2*) and the complemented *ΔMr-OPY2,* (**c**) *ΔMr-STE50* and the complemented *ΔMr-STE50*, and (**d**) *ΔAftf1* and the complemented *ΔAftf1*,. D1 and D2 represent two independent deletion mutants for each gene, and WT is the wild-type strain. The upper portion in the left panel of a, c and d: PCR conducted with the primers Bar-up and the confirmation primer CF-2 (the relative position of all primers are shown in a). PCR products can be obtained only from the deletion mutants of each gene. Lower portion of the left panel in b, c and d: PCR was conducted with primers CF-1 and CF-2; PCR products can be obtained in the WT strain but not in the deletion mutants. Right panel in b, c and d: Confirmation of the complementation of deletion mutants by PCR using the primers ORF-5 and ORF-3. The DNA ladder (DL 10004) was purchased from Generay (Shanghai, China). The uncropped agarose gels in b, c, and d are shown in Supplementary Fig. 15.

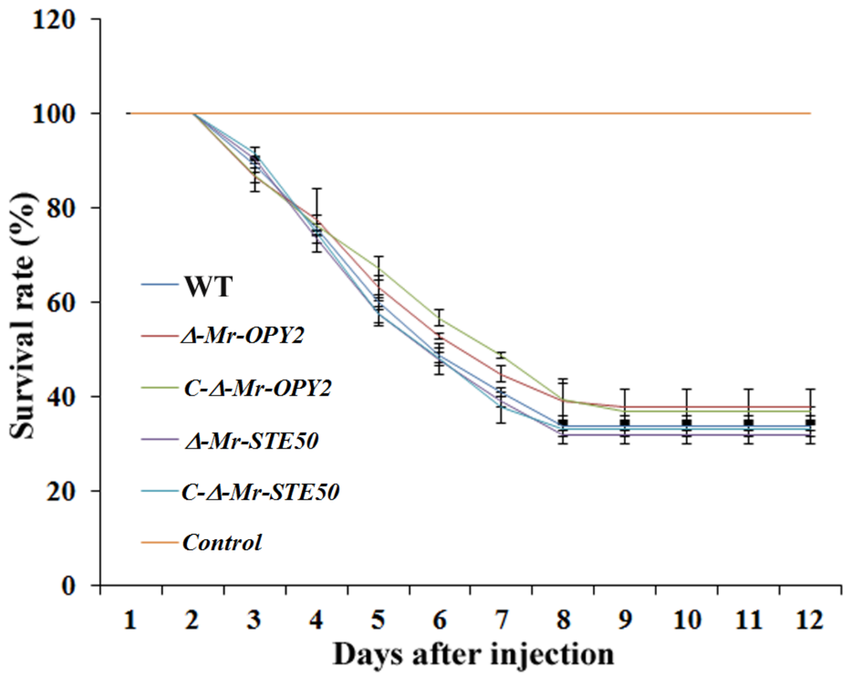

Supplementary Figure 3: Survival curves of *G. mellonella* larvae inoculated by injection of fungal conidia into the insect hemocoel. Three microliters of conidial suspension at 1×10^5 conidia/mL were injected into each larva. Control: the insects were injected with 3μl of the 0.01% TritonX-100 solution that was used for preparation of conidial suspensions. Note: no significant difference ($P > 0.05$, Tukey's test in One-way ANOVA) in virulence was seen between all tested strains. The bioassays were repeated three times with 40 insects per repeat. Data are expressed as the mean ± SE.

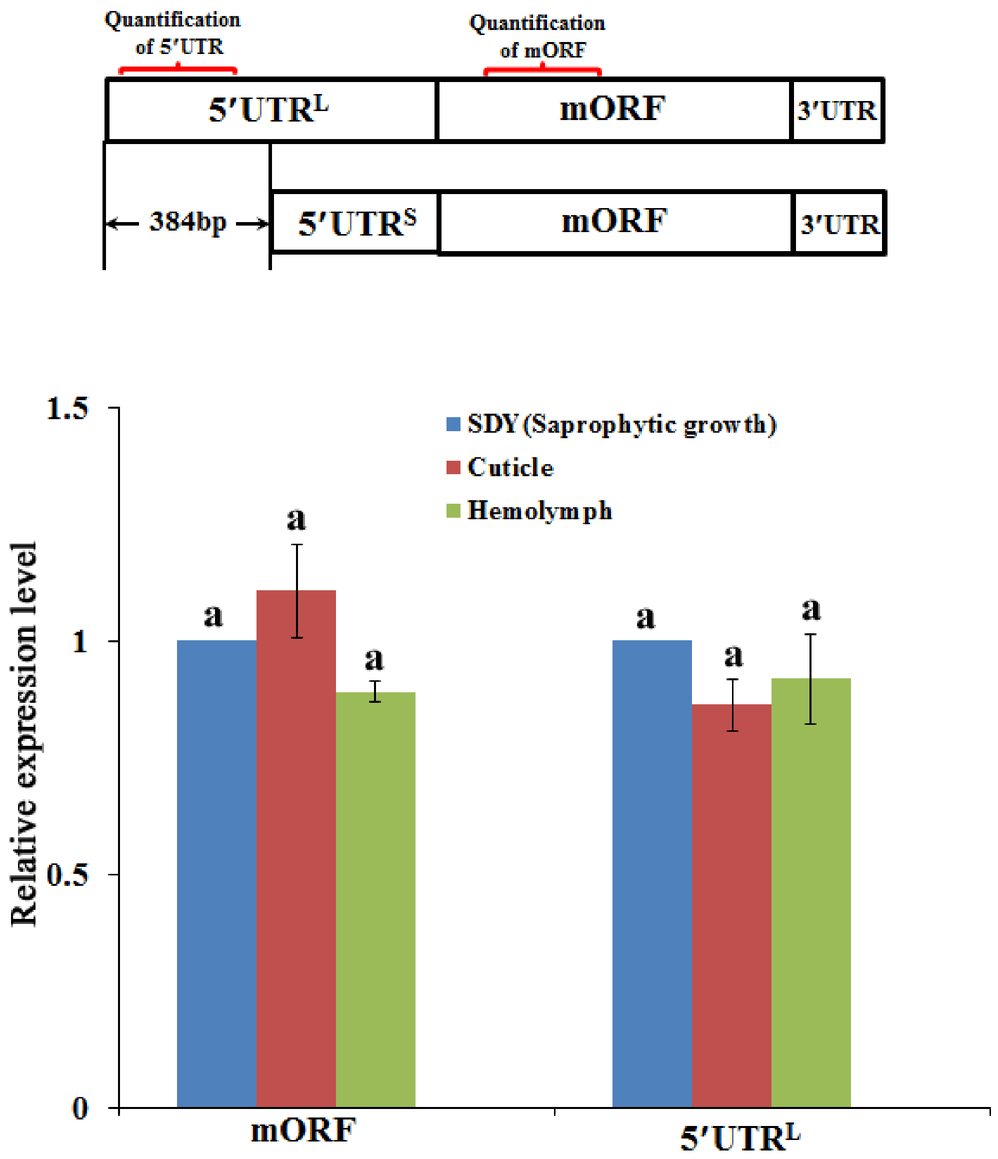

Supplementary Figure 4: qRT-PCR analysis of mORF and 5′UTR^{L} to quantify *Mr-OPY2-L* and *Mr-OPY2-S* transcript levels in total RNA from mycelia grown in SDY or hemolymph, and from germlings differentiating appressoria on locust cuticle. Upper panel: diagram of *Mr-OPY2-L* and *Mr-OPY2-S*. The red brackets are the relative positions of the amplified RNA fragments representing mORF and 5′UTR^{L}. Lower panel: the relative expression level of mORF and 5′UTR^{L} in SDY, hemolymph and cuticle. The expression in SDY (saprophytic growth) was set to 1. The qRT-PCR analyses were repeated three times with three replicates per repeat. Data are expressed as the mean ± SE. Values with different letters are significantly different ($P < 0.05$, Tukey's test in One-way ANOVA).

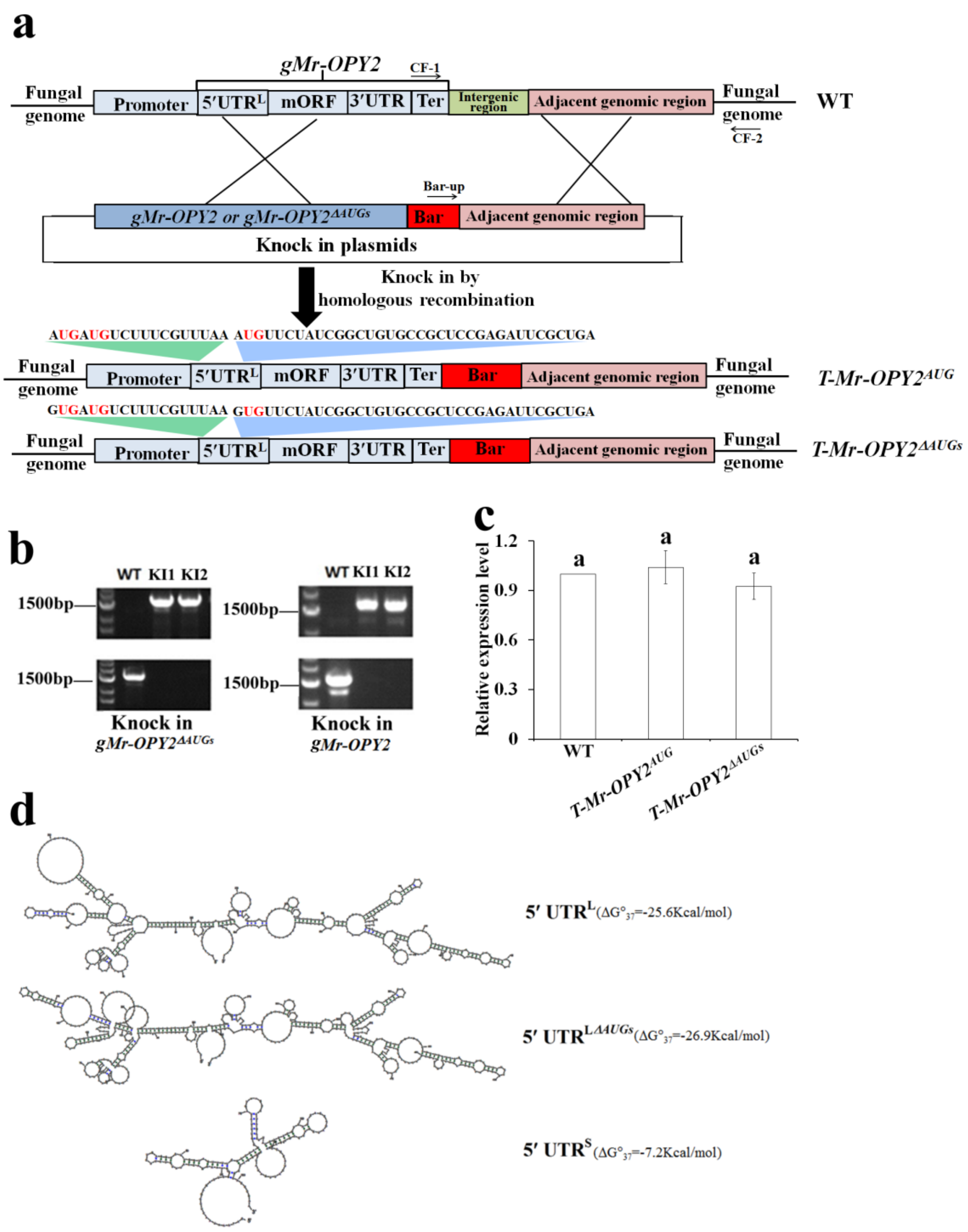

Supplementary Figure 5: Analysis of the impact of uORFs in the 5′UTR of *Mr-OPY2-L* on translation efficiency of the mORF. (**a**) Mutation of the uORFs by changing AUGs into GUGs using site mutagenesis. The uORFs in the genomic clone of the Mr-OPY2 gene (*gMr-OPY2*) were mutated to produce *gMr-OPY2*$^{\Delta AUGs}$ as described in Methods. Upper panel: Diagram showing knock-in of *gMr-OPY2* or *gMr-OPY2*$^{\Delta AUGs}$ in the fungal genome to replace the native *gMr-OPY2*. Note: the intergenic region (green) in the genome was replaced by the *Bar* gene cassette (red) in the knock-in plasmids. Lower panel: two different knock-in strains. *T-Mr-OPY*$^{\Delta AUGs}$ with mutated uORFs and the intergenic region replaced by the *Bar* gene cassette. *T-*

Mr-OPY^{AUG} with only the intergenic region replaced by the *Bar* gene cassette was used as a control to test the effect of deleting the intergenic region on the expression of *Mr-OPY2*. (**b**) PCR confirmation of the knock-in of *gMr-OPY2* or *gMr-OPY2*$^{\Delta AUGs}$ and deletion of the intergenic region in mutants with PPT resistance and without GFP. Left and right panels show the knock-in of *gMr-OPY2*$^{\Delta AUGs}$ and *gMr-OPY2*, respectively. In each panel, KI1 and KI2 represent two independent knock-in strains, and WT is the wild-type strain. PCR was conducted with the primers Bar-up and CF-2 (the relative position of all primers are shown in a), and PCR products can be obtained only from the knock-in strains. Lower panel: PCR was conducted with primers CF-1 and CF-2; PCR products can be obtained from the WT but not from knock-in mutants. The uncropped agarose gels in b are shown in Supplementary Fig. 15 (**c**) Relative expression level of *Mr-OPY2*'s mORF in WT and two knock-in strains. The expression in WT was set to 1. The qRT-PCR analyses were repeated three times with three replicates per repeat. Data are expressed as the mean ± SE. Values with different letters are significantly different ($P < 0.05$, Tukey's test in One-way ANOVA). (**d**) Secondary structures of the *5′UTR*^{S} (the 5′UTR of the short mRNA), *5′UTR*^{L} (the 5′UTR of the long mRNA) and *5′UTR*$^{L\Delta AUGs}$ (the 5′UTR of the mutated long mRNA with the uORFs changed).

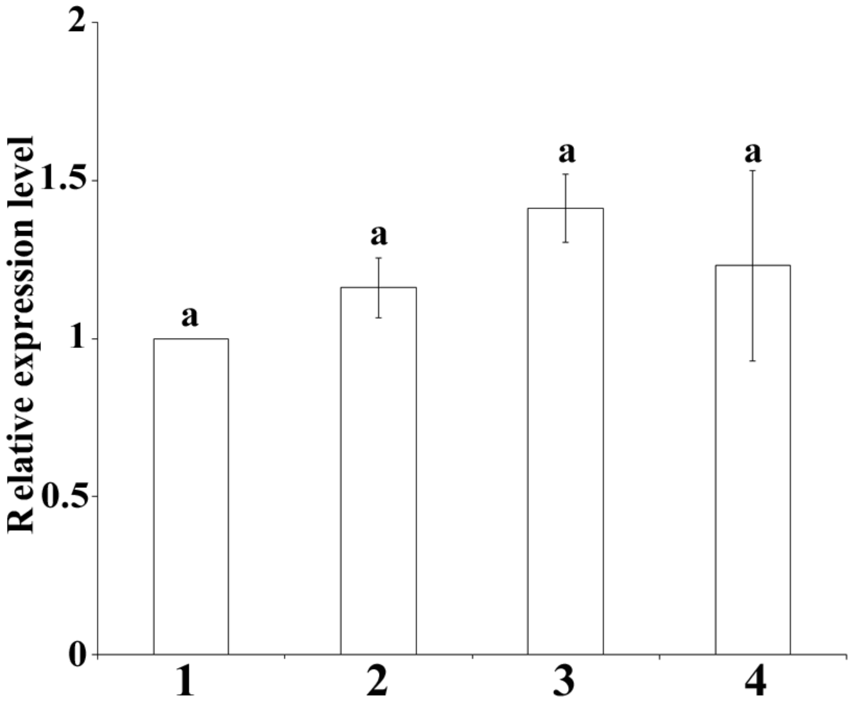

Supplementary Figure 6: Relative expression level of *Mr-OPY2* mORF in the WT and strains with expression of either *Mr-OPY2-L*, *Mr-OPY2-S* or mutated *Mr-OPY2-L* driven by the *M. acridum Pgpd* promoter (its 5′UTR excluded). 1) wild type strain; 2) *ΔMr-OPY2:Mr-OPY2-S*; 3) *ΔMr-OPY2:Mr-OPY2-L*, and 4) $\Delta Mr\text{-}OPY2{:}Mr\text{-}OPY2\text{-}L^{\Delta AUGs}$. The expression in WT was set to 1. The qRT-PCR analyses were repeated three times with three replicates per repeat. Data are expressed as the mean ± SE. Values with different letters are significantly different ($P < 0.05$, Tukey's test in One-way ANOVA).

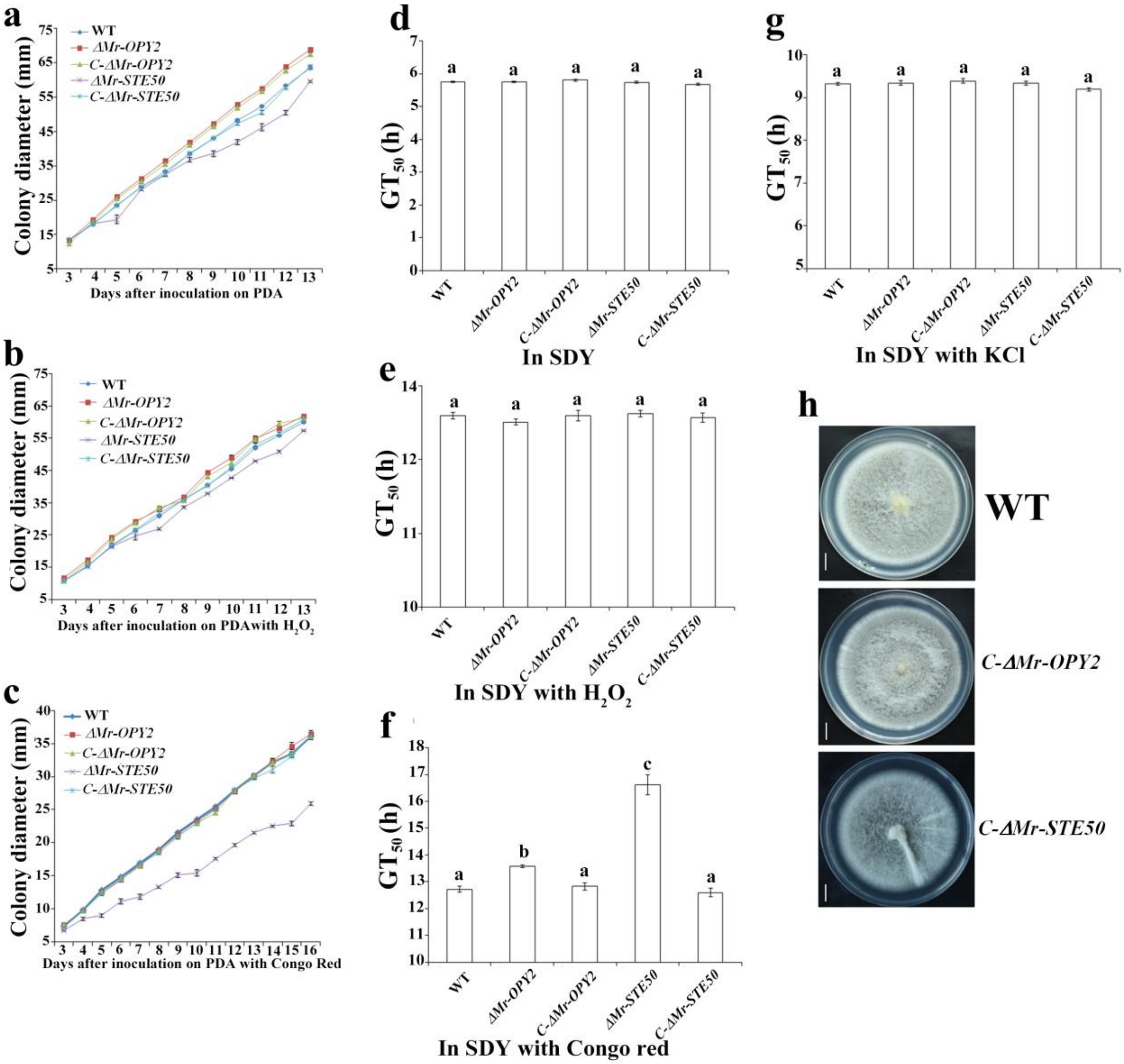

Supplementary Figure 7: Growth and germination rates of WT, *ΔMr-OPY2*, *ΔMr-STE50* (and their respective complemented strains) with or without abiotic stresses. Growth curves, measured as diameters of colonies, on (**a**) PDA plates, (**b**) PDA supplemented with 0.01% H_2O_2, (**c**) PDA supplemented with 1mg/ml Congo Red. Germination rates, shown by GT_{50} (time taken for 50% of conidia to germinate), in (**d**) SDY medium, (**e**) SDY plus 0.01% H_2O_2, (**f**) SDY plus 1mg/ml Congo Red, (**g**) SDY plus 0.75M KCl. All growth and germination assays were repeated three times with three replicates per repeat. Data are expressed as the mean ± SE. Values with different letters are significantly different ($P < 0.05$, Tukey's test in one-way ANOVA). Note: Significant differences in growth rate on PDA with Congo Red was observed between *ΔMr-STE50* and other strains (**c**); significant difference in germination rate in SDY plus Congo Red was also seen between *ΔMr-OPY2*, *ΔMr-STE50* and other strains (**f**). (**h**) Colony phenotype of WT, the complemented strains of *ΔMr-OPY2* and *ΔMr-STE50* on PDA plates. Pictures were taken 18 days after inoculation. Scale bar represents 10mm Images are representative of at least three independent experiments.

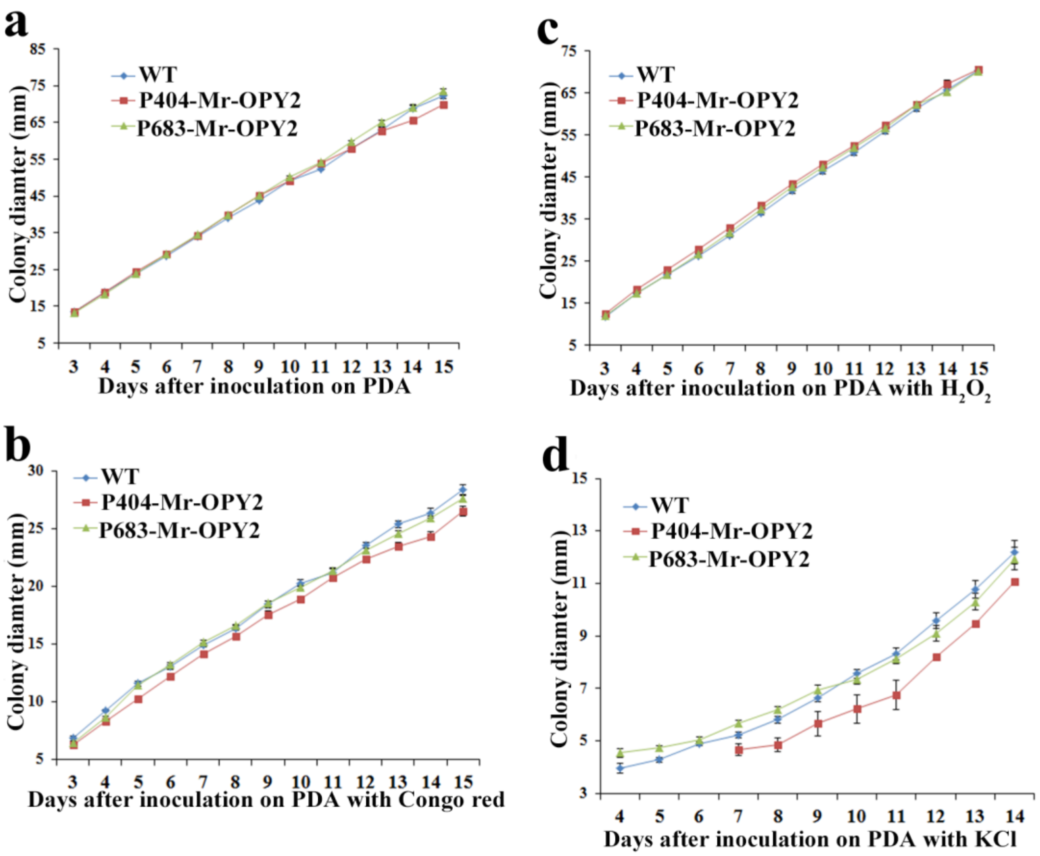

Supplementary Figure 8: Growth curves of WT, P404-Mr-OPY2 and P683-Mr-OPY2 strains that produce different levels of Mr-OPY2 protein. Growth curves measured from the diameter of colonies produced on (**a**) PDA plates, (**b**) PDA supplemented with 0.01% H_2O_2, (**c**) PDA supplemented with Congo Red (1mg/ml) and (**d**) PDA plus 0.75M KCl. All growth assays were repeated three times with three replicates per repeat. Data are expressed as the mean ± SE. Note: no significant difference in growth was observed between the three strains at the four conditions ($P < 0.05$, Tukey's test in one-way ANOVA).

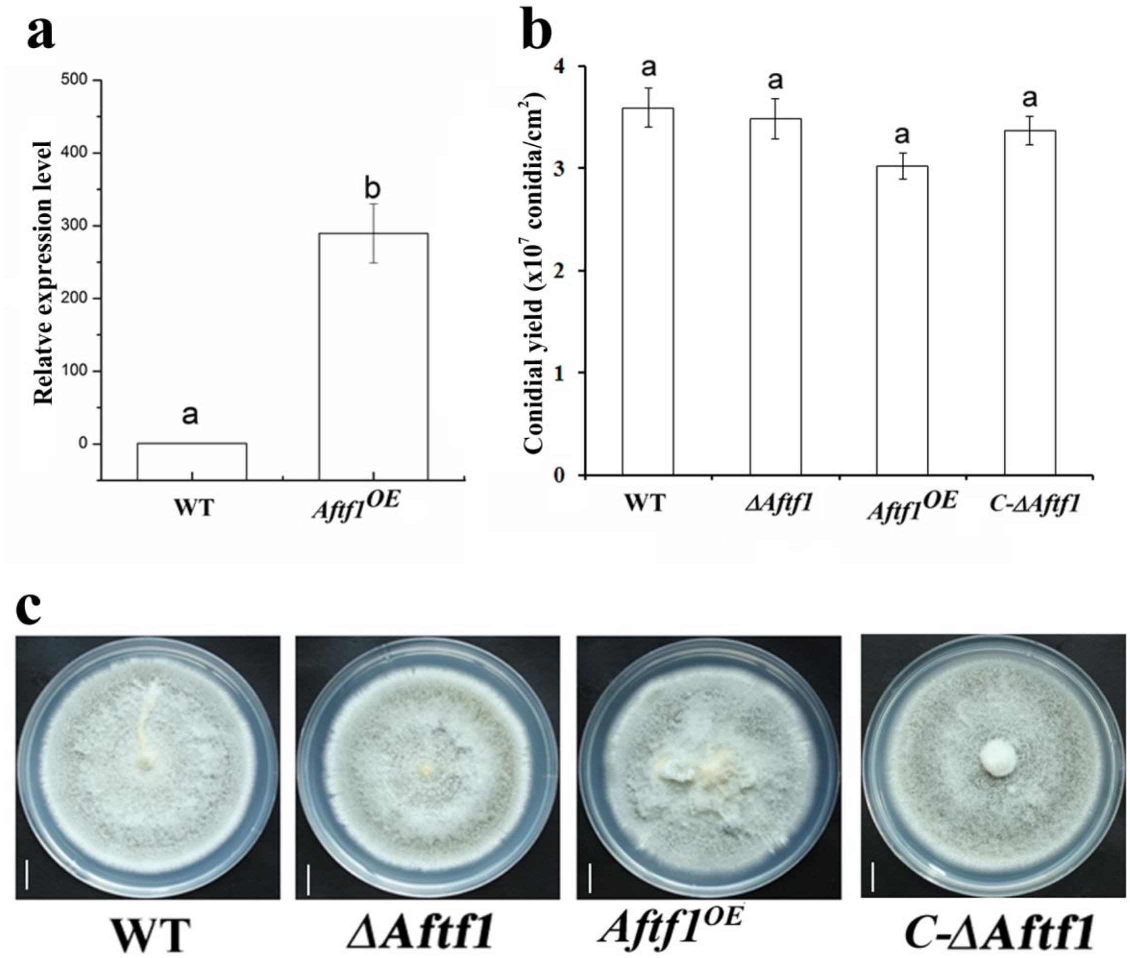

Supplementary Figure 9: Colony phenotype and conidial yields of WT, the deletion *Aftf1* mutant (*ΔAftf1*), the complemented *ΔAftf1* (*C-ΔAftf1*), and the strain overexpressing *Aftf1* (*Aftf1*^{OE}). (**a**) qRT-PCR confirmation of overexpression of the *Aftf1* gene driven by the constitutive promoter (*Ptef)* of the translation elongation factor gene from *A. pullulans*. (**b**) Conidial yields and (**c**) colony phenotype of WT, *ΔAftf1*, *C-ΔAftf1*and *Aftf1*^{OE}. The qRT-PCR analyses and quantification of conidial yield were repeated three times with three replicates per repeat. Data are expressed as the mean ± SE. In a and b, values with different letters are significantly different (n = 3 for the qRT-PCR analyses, n = 9 for the conidial yield assays, $P < 0.05$, one-way ANOVA). In c, pictures were taken 18 days after inoculation; images are representative of at least three independent experiments. Scale bar represents 10mm.

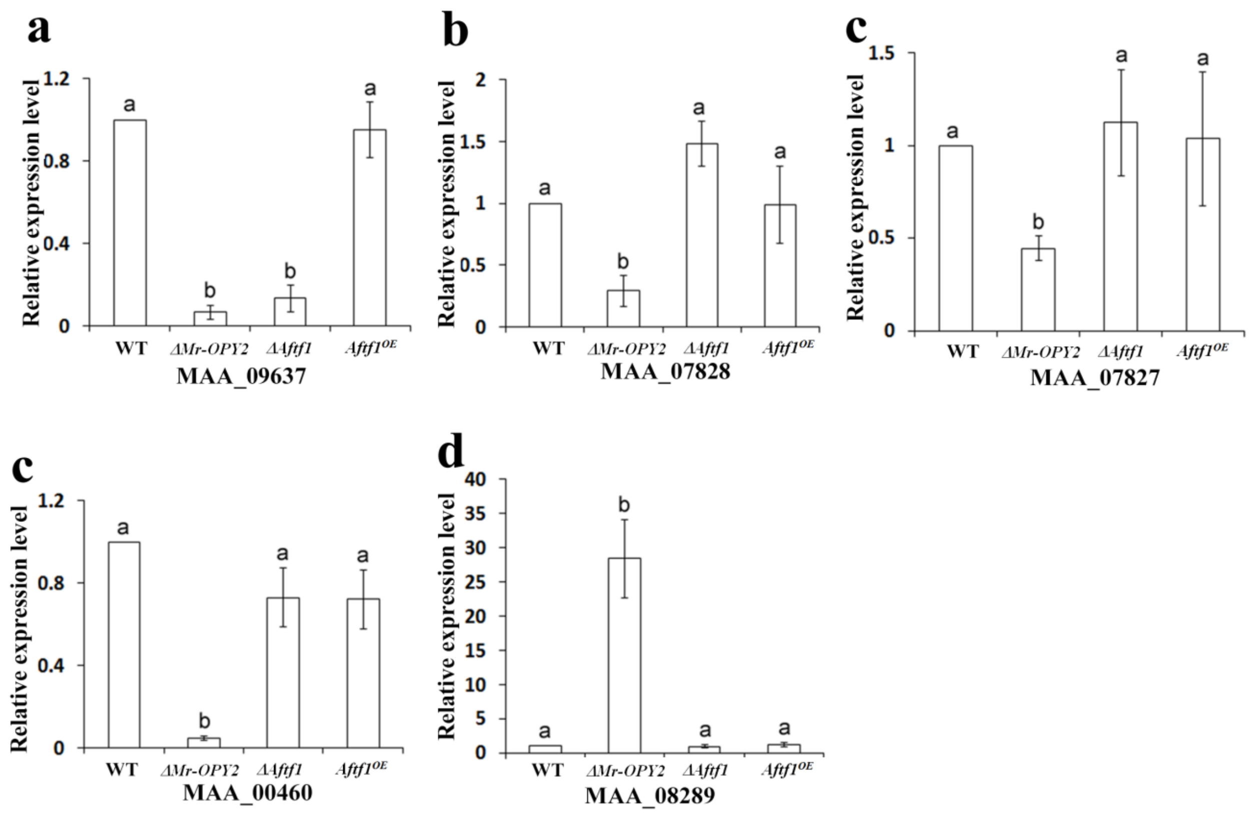

Supplementary Figure 10: qRT-PCR analysis of five genes in the WT, the *Mr-OPY2* deletion mutant (*ΔMr-OPY2*), the *Aftf1* deletion mutant (*ΔAftf1*) and the strain overexpressing *Aftf1* (*Aftf1*^{OE}). The expression in WT was set to 1. The qRT-PCR analyses were repeated three times with three replicates per repeat. Data are expressed as the mean ± SE. Values with different letters are significantly different ($P < 0.05$, Tukey's test in one-way ANOVA).

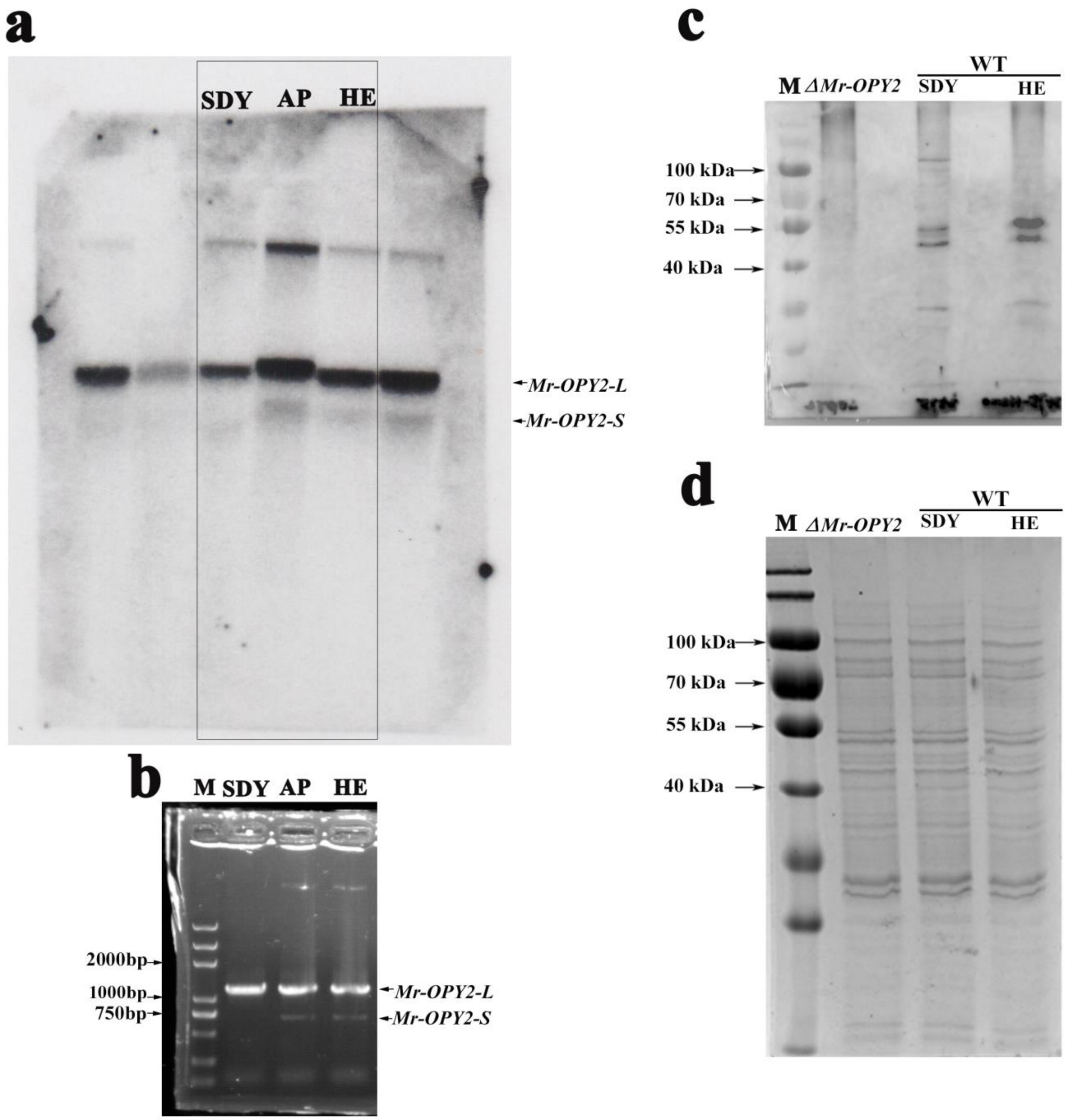

Supplementary Figure 11: Uncropped images of Northern blots, Western blots, SDS-PAGE and agarose gels shown in Fig. 2. (a) The Northern blot (the boxed part) of *Mr-OPY2* shown in Fig.2a. (b) The agarose gel shown in Fig. 2b. (c) The Western blot shown in Fig. 2d (upper panel). (d) The SDS-PAGE gel shown in Fig. 2d (lower panel).

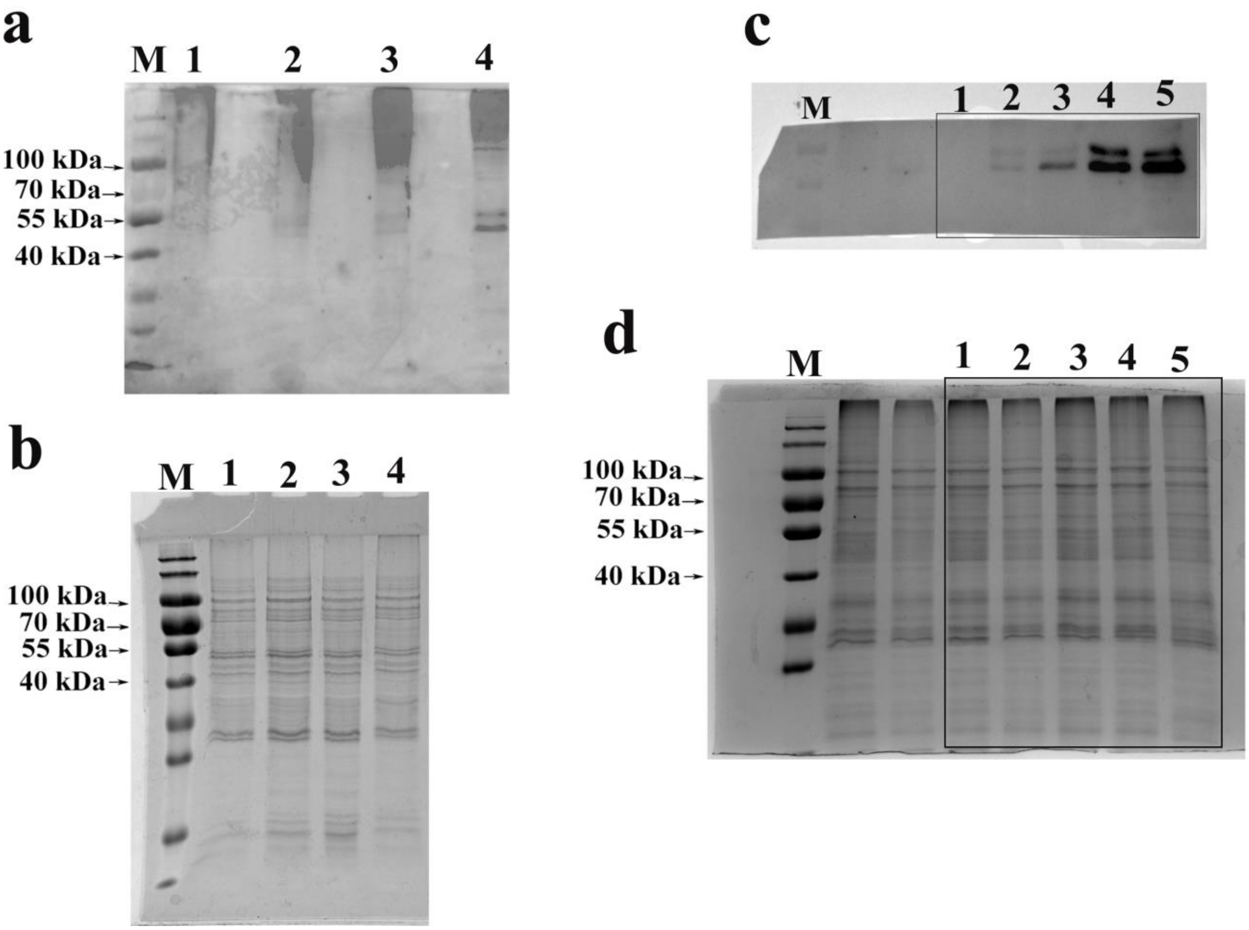

Supplementary Figure 12: Uncropped images of Western blots and SDS-PAGE gels shown in Fig. 3. (a) The Western blot shown in Fig. 3a (middle panel). (b) The SDS-PAGE gel shown in Fig. 3a (lower panel). (c) The Western blot (the boxed part) shown in Fig. 3b (middle panel). (d) The SDS-PAGE gel (the boxed part) shown in Fig. 3b (lower panel).

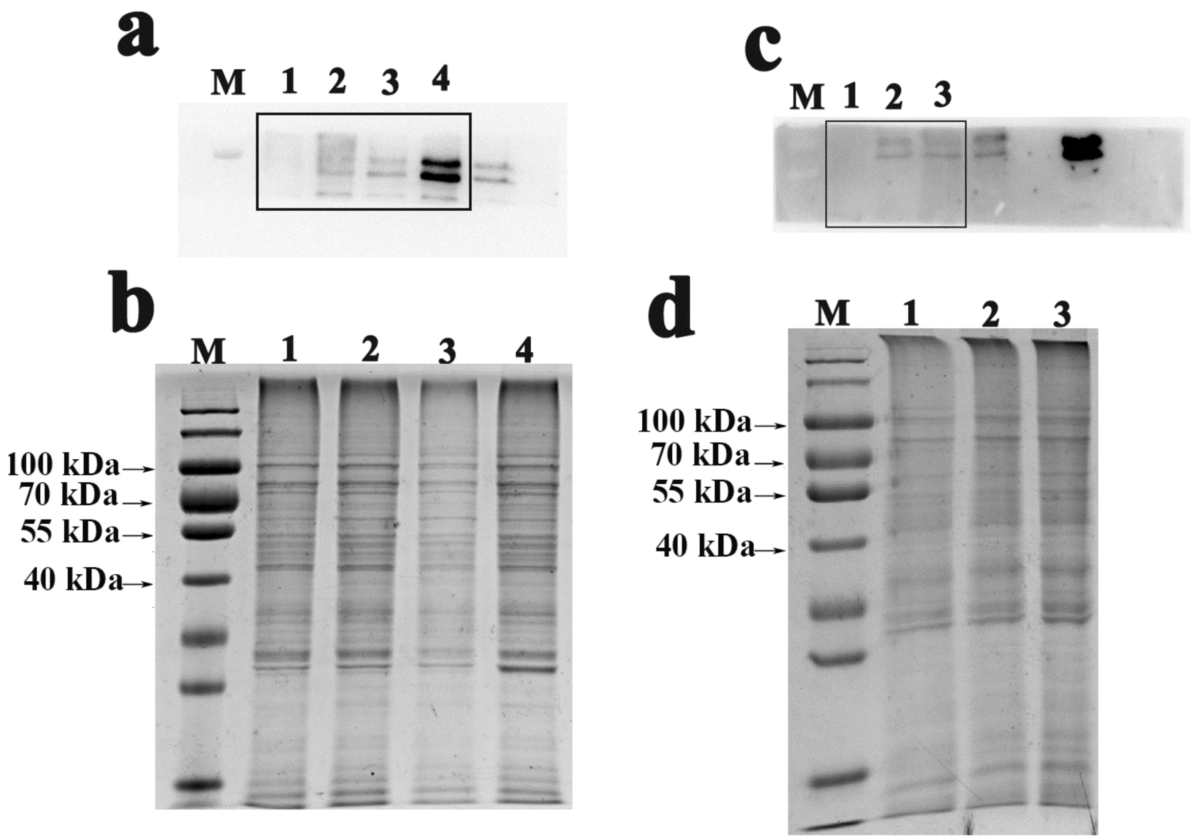

Supplementary Figure 13: Uncropped images of Western blots and SDS-PAGE gels shown in Fig. 4 and Fig. 5. (a) The Western blot (the boxed part) shown in Fig. 4a (middle panel). (b) The SDS-PAGE gel shown in Fig. 4a (right panel). (c) The Western blot (the boxed part) shown in Fig. 5c (upper panel). (d) The SDS-PAGE gel shown in Fig. 5c (lower panel).

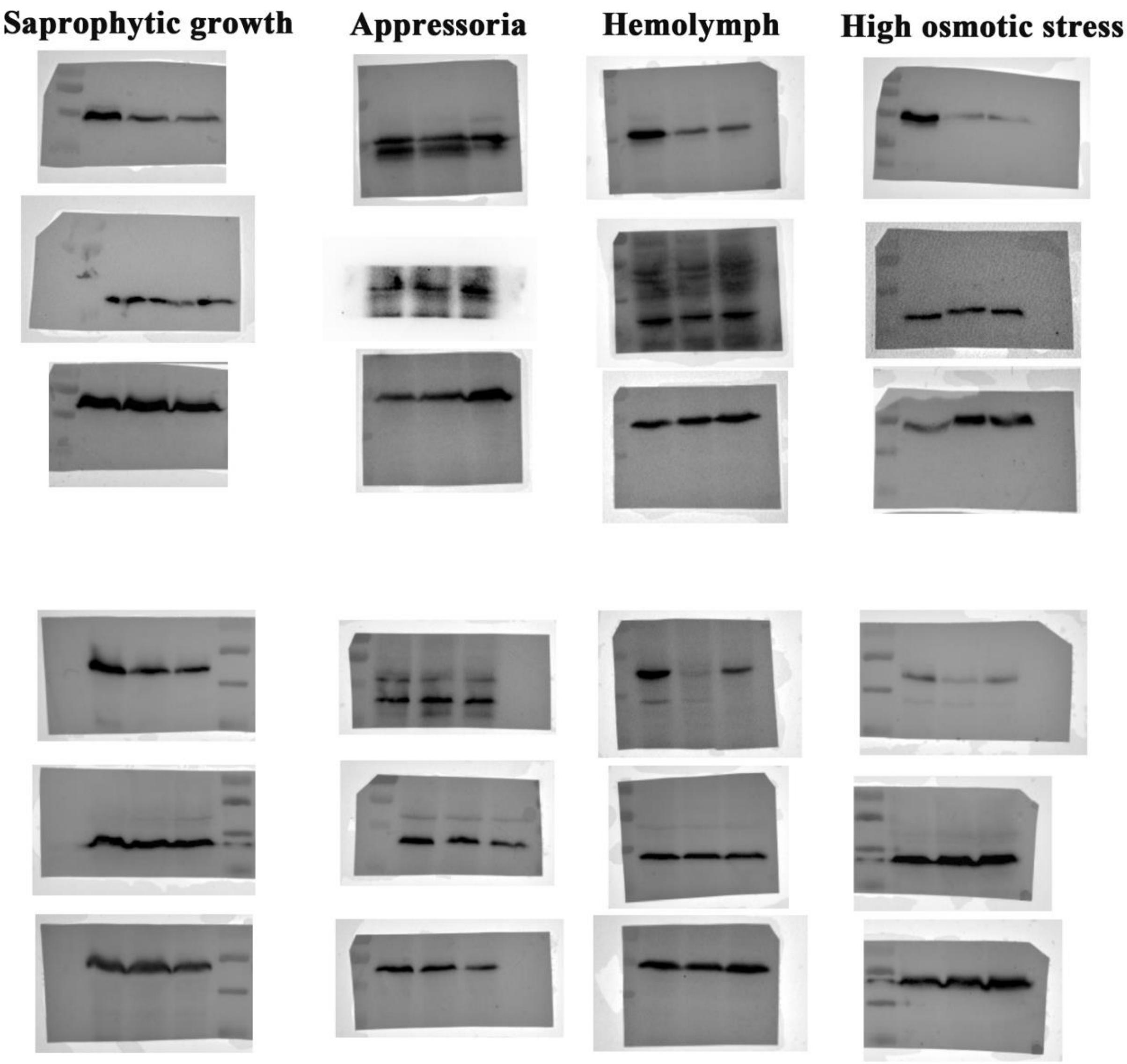

Supplementary Figure 14: Uncropped images of Western blots shown in Fig. 6.

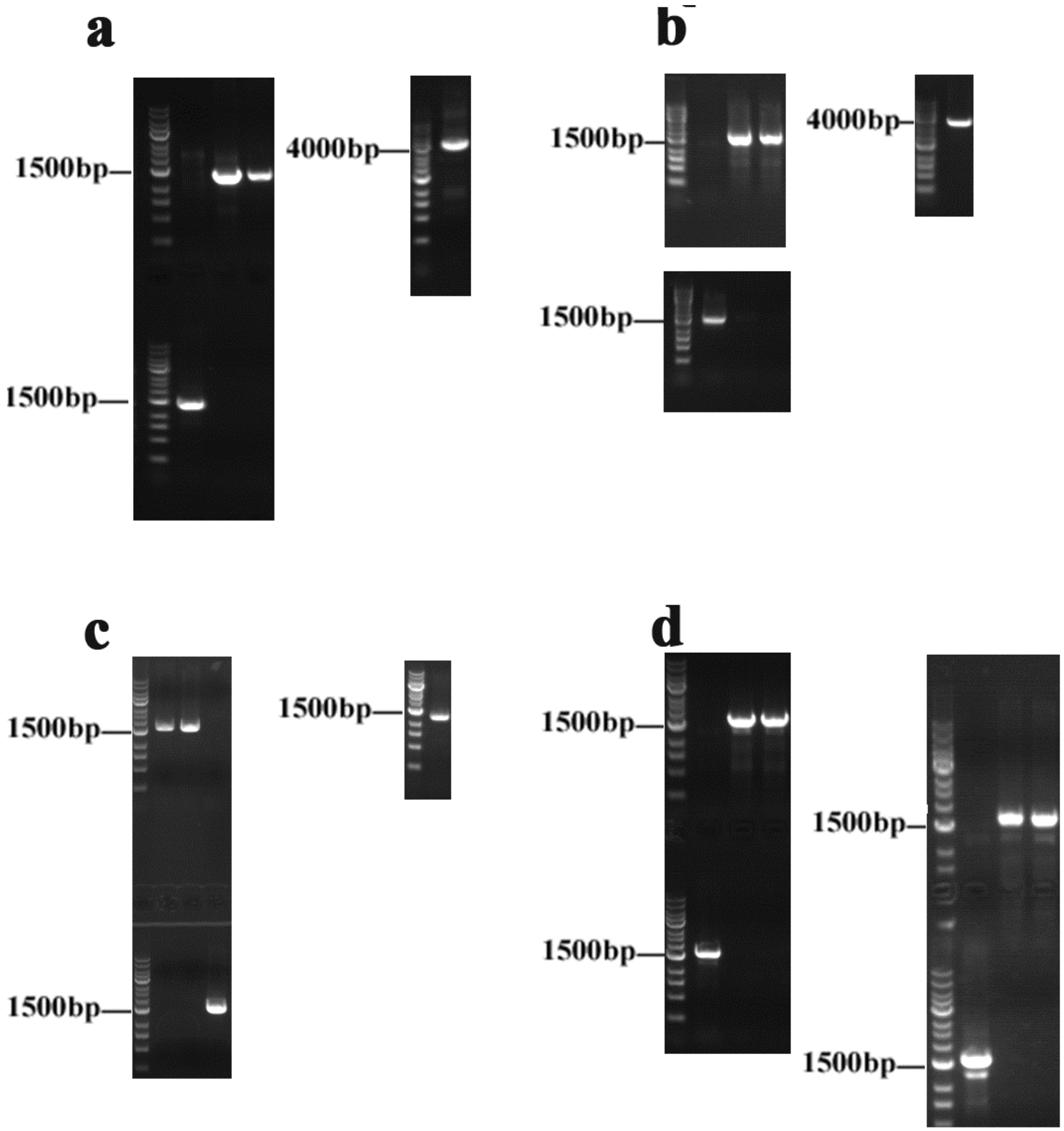

Supplementary Figure 15: Uncropped images of agarose gels shown in the supplementary figures. (a) The agarose gels shown in Supplementary Fig. 2b, (b) Supplementary Fig. 2c, (c) Supplementary Fig. 2d, (d) Supplementary Fig. 5b.

Supplementary Table 1: Proteins used in the phylogenetic analysis shown in Supplementary Fig. 1

Phylum	Species	Accession numbers
Ascomycota	*Metarhizium robertsii*	EFZ01771.1
	Metarhizium anisopliae	KFG88145.1
	Metarhizium brunneum	KID76312.1
	Metarhizium guizhouense	KID87777.1
	Metarhizium majus	KID98886.1
	Metarhizium acridum	EFY90059.1
	Metarhizium album	KHN96677.1
	Pochonia chlamydosporia	OAQ61876.1
	Metarhizium rileyi	OAA42181.1
	Ustilaginoidea virens	KDB11227.1
	Aschersonia aleyrodis	KZZ89148.1
	Claviceps purpurea	CCE32422.1
	Cordyceps confragosa	OAR01272.1
	Cordyceps confragosa	OAA77151.1
	Beauveria bassiana	EJP66177.1
	Cordyceps militaris	EGX93710.1
	Isaria fumosorosea	OAA55374.1
	Drechmeria coniospora	ODA80581.1
	Hirsutella minnesotensis	KJZ76340.1
	Ophiocordyceps unilateralis	KOM22650.1
	Ophiocordyceps sinensis	EQK99523.1
	Trichoderma harzianum	KKP04068.1
	Neonectria ditissima	KPM34106.1
	Fusarium avenaceum	KIL96414.1
	Fusarium oxysporum	EXM32794.1
	Fusarium graminearum	ESU16791.1
	Fusarium pseudograminearum	EKJ67761.1
	Neurospora crassa	EAA32104.2
	Magnaporthe oryzae	EHA52440.1
	Verticillium dahliae	EGY18258.1
	Colletotrichum incanum	KZL65049.1
	Colletotrichum higginsianum	OBR07757.1
	Aspergillus nidulans	XP_660126.1
	Aspergillus fumigatus	XP_754185.1
Basidiomycota	*Ustilago maydis*	XP_011390299.1
	Kalmanozyma brasiliensis	XP_016291896.1
	Pseudozyma hubeiensis	XP_012192749.1
	Sporisorium reilianum	CBQ68277.1
Yeasts	*Candida albicans*	XP_003958628.1
	Zygosaccharomyces bailii	CDF89653.1
	Kazachstania naganishii	CCK68385.1
	Saccharomyces cerevisiae	AJV94457.1
	Naumovozyma dairenensis	XP_003667903.1
	Kazachstania africana	XP_003958628.1

Supplementary Table 2: Primers used in this study

Primer	Sequence	Usage
DMr-OPY2 -5-1	GGTCTAGATGAGACCTGCACTGGCTC	Disruption of *Mr-OPY2*
DMr-OPY2 -5-2	GGTCTAGACATATGTACCCCTCTTGC	
DMr-Opy2 -3-1	GGACTAGTAGTCGCGGCGTCCAACATG	
DMr-Opy2- 3-2	GGACTAGTACCTCGCGGACCATCGGC	
DMr-Ste50 5-1	GGTCTAGAGCCTCCTGCCCCCAGGTG	Disruption of *Mr-Ste50*
DMr-Ste50 5-2	GGTCTAGACGACCAGGGCTTCGCCAAC	
DMr-Ste50 3-1	GGTCTAGATTGCTGAACAAACGCTGG	
DMr-Ste50 3-2	GGTCTAGACCAATTTCTGCCGCAGAC	
DAftf1-5-1	GGGGACAGCTTTCTTGTACAAAGTGGAATGGAGCTCGCGATGAAC	Disruption of *Aftf1*
DAftf1-5-2	GGGGACTGCTTTTTTGTACAAACTTGTGAAGCAATCGACCAGAC	
DAftf1-3-1	GGGGACAACTTTGTATAGAAAAGTTGTTGCATAGTTGCTATCAAG	
DAftf1-3-2	GGGGACAACTTTGTATAATAAAGTTGTCGCTGTCCATCGTCTTG	
Bar-up	CGCCTGGACGACTAAACC	Confirmation of gene disruptions
Bar-down	TCAGCCTGCCGGTACCGC	
DMr-OPY2-CF-1	GCAGACAATTTGATAACC	Confirmation of the disruption of
DMr-OPY2-CF-2	ATACCATTGAGTTCAGTC	*Mr-OPY2*
DMr-Ste50-CF-1	CCCCAAGAACGCTTCACC	Confirmation of the disruption of
DMr-Ste50-CF-2	CATCGGAATGGTAAAGTG	*Mr-Ste50*
DAftf1-CF-1	TGTTAGAGTCGGCAGAG	Confirmation of the disruption of
DAftf1-CF-2	ACATCTTTGACCAGAAG	*Aftf1*
Mr-OPY2 -5	GGACTAGTTCACTGTTGGACTCTGAG	Cloning the genomic clone of
Mr-OPY2 -3	GGCCCGGGCCCTTCTTTCATCCATTC	*Mr-OPY2* for complementation
Mr-Ste50-5	GGTTTAAAACATACACACTGGTAGG	Cloning the genomic clone of
Mr-Ste50-3	GGTCTAGAAAGCTCGTAGCAACTGC	*Mr-Ste50* for complementation
Aftf1-5	GGTTTAAACTGGTATGGTGAAGATG	Cloning the genomic clone of
Aftf1-3	GGACTAGTGATGATGATCATGATGC	*Aftf1* for complementation
Mero-Bck1-ORF-5	CCATCGATATGTATCAAGGCGGCCA	Cloning the ORF of Mero-Bck1
Mero-Bck1-ORF-3	CCCTAGCAGCTACGAGATCTTGTATG	
Mero-Ssk2-ORF-5	CCTTTAAAATGTCGGACCCGTCTCC	Cloning the ORF of *Mero-SSK2*
Mero-Ssk2-ORF-3	CCGGATCCTTATGATCCATCCGGCA	
Mero-Ste11-ORF-5	CCTTTAAAATGGCGATGCTCTCGTC	Cloning the ORF of *Mero-STE11*
Mero- Ste11-ORF-3	CCTTTAAACTAGGTCATGTGCGAGA	
Mr-OPY2-ORF-5-1	GGGAATTCATGATTGGCCAAACACC	Cloning the ORF of *Mr-OPY2*
Mr-OPY2-ORF-3-2	GGAGATCTTTATTGTGAGCAGGTGACG	
Mr-Ste50-ORF-5	GGGAATTCATGATGAGCTTCGATGGC	Cloning the ORF of *Mr-STE50*
Mr-Ste50-ORF-3	GGGGATCC TCATATAATGCCTCCAGG	
ACT-F	TCCTGACGGTCAGGTCATC	reference for qRT-PCR
ACT-R	CACCAGACATGATGTTG	
TEF-F	CTGGTACAAGGGTTGGGAGA	reference for qRT-PCR
TEF-R	TACACATCCTGGAGGGGAAG	
Mr-OPY2-ORF-5-F	CCACACAGTTCATTCCATCG	q-RT-PCR of Mr-OPY2-ORF
Mr-OPY2-ORF-3-R	TCCTGGCTACTATGACGGCT	
Mr-OPY2-UTR-5-F	GCCAAGGTCGATGATGTCTT	q-RT-PCR of 5’UTR^{L}
Mr-OPY2-UTR-3-R	GAAGCGAAATAGCAACGGAG	
Aftf1-ORF-F	CCGACATCAAGCAGGACATG	q-RT-PCR of *Aftf1*
Aftf1-ORF-R	CTTGTTCTTGATGCCTCGGG	
MAA_00460-F	CATAAGTGTCGGCATGCTCC	q-RT-PCR for MAA_00460
MAA_00460-R	AACCGTTCTTTGACCTTGCC	
MAA_07827-F	TTGGTCTTTGGTAGCCGACT	q-RT-PCR for MAA_07827
MAA_07827-R	CTCTTCGGCGAGCGATACTA	
MAA_07828-F	CGCTCTCTATGCTTCGCTTC	q-RT-PCR for MAA_07828
MAA_07828-R	TCTTATCCTCGTGTCGGCTC	
MAA_08289-F	CATTGTCTTCGCTGCCTCTC	q-RT-PCR for MAA_08289
MAA_08289-R	GACATCGCGGATAATGGCTG	
MAA_09637-F	GTCGTAGAAGCAGCCTTTGG	q-RT-PCR for MAA_09637
MAA_09637-R	GCATCTTGCATCTGCTGGTA	
Mr-OPY2-5’UTR^{L}-5	GGGAATTCTGAGACCTGCACTGGCTC	
Mr-OPY2-5’UTR^{S}-5	GGGAATTCCTCCGAGATTCGCTGAAAC	Cloning the long and short transcripts
Mr-OPY2-3’UTR-3	GGCCCGGGAAATGTCAGCCACACCA	
MAC_Pgpd683-n5’UTR	GGGAATTCAGCGGGAAGAGAAAACA	Cloning P683 without its own5’UTR

MAA_pgpd-404-5	GGCCCGGGACTTGACCAGTTTTGCG	Cloning P404 and P683
MAA_pgpd-683-5	GGCCCGGGCATTCGATTCCTCATCA	
MAA_pgpd-404/683-3	CCGGATTCTTTGCGTGTGTGTATAT	
3'RACE-GSP-outer	CTACAATCTACGGCAAGCAGGCTC	3'RACE
3'RACE-GSP-inner	CCAAGCCAACTGTTGTCAGCGTTAG	
5'-RLM-RACE-outer	AGATGCCCGAGTACGTC	5'-RLM-RACE
5'-RLM-RACE-inner	CGGCTGCCCTCAGAGTG	
KIOPY2_CF1	TACTGGGTATCAGGGCAC	Confirmation of knock in strains
KIOPY2_CF2	GATACCTTCTGCTGAAG	
gMr-OPY2-F	TGAGACCTGCACTGGCTCTC	
gMr-OPY2-R	GCGCCGTAACCTTCGTAACC	
gMr-OPY2$^{\Delta AUG167}$-F	CTGTGTCATTGCATATCTTGTTACGGGTGTTCTATCGGCTGTGCCGCTC	Mutate uORFs in 5'UTR of Mr-OPY2-L
gMr-OPY2$^{\Delta AUG167}$-R	ATCTCGGAGCGGCACAGCCGATAGAACACCCGTAACAAGATATGCAATGACACA	
gMr-OPY2$^{\Delta AUGs}$-F	CTTCACTTCATCCAGTCGCCAAGGTCGGTGGTGTCTTTCGTTTAAAAAAAAA	
gMr-OPY2$^{\Delta AUGs}$-R	TTTGGTGTTTTTTTTTTAAACGAAAGACACCACCGACCTTGGCGACTGGATGA	
Aftf1-ORF5	GGGAATTCATGGCCGACATCAAGCA	Overexpressing *Aftf1*
Aftf1-ORF3	GGATATCCTACTCTATGGCAATTC	
